# Global sales of oral antibiotics formulated for children

**DOI:** 10.2471/BLT.19.235309

**Published:** 2020-05-08

**Authors:** Grace Li, Charlotte Jackson, Julia Bielicki, Sally Ellis, Yingfen Hsia, Mike Sharland

**Affiliations:** a Paediatric Infectious Diseases Research Group, Institute of Infection and Immunity, St George’s, University of London, Cranmer Terrace, London, SW17 0QT, England.; b Global Antibiotic Research & Development Partnership, Geneva, Switzerland.; c School of Pharmacy, Queen’s University Belfast, Belfast, Northern Ireland.

## Abstract

**Objective:**

To investigate international consumption patterns of child-appropriate oral formulations of antibiotics by formulation type, with a focus on dispersible tablets, using data from a global sales database.

**Method:**

Antibiotic sales data for 2015 covering 74 countries and regional country groups were obtained from the MIDAS^®^ pharmaceutical sales database, which includes samples of pharmacy wholesalers and retailers. The focus was on sales of child-appropriate oral formulations of Access antibiotics in the 2017 World Health Organization’s *WHO Model list of essential medicines for children*. Sales volumes are expressed using a standard unit (i.e. one tablet, capsule, ampoule or vial or 5 mL of liquid). Sales were analysed by antibiotic, WHO region and antibiotic formulation.

**Findings:**

Globally, 17.7 billion standard units of child-appropriate oral antibiotic formulations were sold in 2015, representing 24% of total antibiotic sales of 74.4 billion units (both oral and parenteral) in the database. The top five child-appropriate Access antibiotics by sales volume were amoxicillin, amoxicillin with clavulanic acid, trimethoprim–sulfamethoxazole, cefalexin and ampicillin. The proportion of the top five sold for use as a syrup varied between 42% and 99%. Dispersible tablets represented only 22% of all child-appropriate oral formulation sales and made up only 15% of sales of 10 selected Access antibiotics on the model list for children.

**Conclusion:**

Globally most child-appropriate oral antibiotics were not sold as dispersible tablets in 2015, as recommended by WHO. There is a clear need for novel solid forms of antibiotics suitable for use in children.

## Résumé

**Objectif:**

Étudier la consommation internationale d'antibiotiques à usage oral destinés aux enfants en fonction du type de formulation, en mettant l'accent sur les comprimés dispersibles. Et ce, en utilisant les données issues d'une base de données mondiale relative aux ventes.

**Méthodes:**

Les informations sur la vente d'antibiotiques en 2015 dans 74 pays et groupes régionaux de pays proviennent de la base de données de ventes pharmaceutiques MIDAS^®^, qui inclut des échantillons de grossistes et détaillants de produits pharmaceutiques. La priorité était accordée aux antibiotiques à usage oral destinés aux enfants dont l'accessibilité est essentielle (Access), conformément à l'édition 2017 de la *Liste modèle des médicaments essentiels destinés à l'enfant* de l'Organisation mondiale de la Santé (OMS). Les volumes de vente sont exprimés selon une unité de mesure standard (c'est-à-dire un comprimé, une capsule, une ampoule ou flacon, ou 5 ml de liquide). Les ventes ont été analysées pour chaque antibiotique, région OMS et formulation d'antibiotique.

**Résultats:**

À l'échelle mondiale, 17,7 milliards d'unités standard d'antibiotiques à usage oral destinés aux enfants ont été écoulées en 2015, ce qui équivaut à 24% du volume total de vente d'antibiotiques (74,4 milliards d'unités, tant par voie orale que parentérale) que contient la base de données. Les cinq principaux antibiotiques Access administrés aux enfants étaient l'amoxicilline, l'amoxicilline + acide clavulanique, le sulfaméthoxazole + triméthoprime, la céfalexine et l'ampicilline. Le pourcentage de vente pour ces cinq antibiotiques, commercialisés sous forme de sirop, était compris entre 42% et 99%. Les comprimés dispersibles ne représentaient que 22% de l'ensemble des formulations à usage pédiatrique, et seulement 15% des ventes pour les 10 antibiotiques Access sélectionnés dans la liste modèle.

**Conclusion:**

La plupart des antibiotiques administrés par voie orale aux enfants n'étaient pas vendus sous forme de comprimés dispersibles en 2015 dans le monde, contrairement aux recommandations de l'OMS. Il est donc nécessaire de développer de nouvelles formes solides d'antibiotiques, adaptées à un usage pédiatrique.

## Resumen

**Objetivo:**

Investigar los patrones de consumo internacional de las formulaciones pediátricas orales apropiadas de los antibióticos según el tipo de formulación, con énfasis en los comprimidos dispersables y mediante el uso de la información de una base de datos de ventas mundiales.

**Métodos:**

Los datos sobre las ventas de antibióticos correspondientes a 2015, que abarcan 74 países y grupos regionales de países, se obtuvieron de la base de datos sobre las ventas de los productos farmacéuticos MIDAS^®^, que incluye muestras de mayoristas y de minoristas de farmacias. El enfoque se centró en la venta de las formulaciones pediátricas orales apropiadas de los antibióticos del Grupo de acceso (*Access*) de la *Lista modelo de medicamentos pediátricos esenciales* de 2017 de la Organización Mundial de la Salud (OMS). Las cantidades que se venden se expresan en una unidad estándar (es decir, un comprimido, una cápsula, una ampolla, un vial o 5 ml de líquido). Las ventas se analizaron por antibiótico, región de la OMS y formulación del antibiótico.

**Resultados:**

A nivel mundial, se vendieron 17 700 millones de unidades estándar de formulaciones pediátricas apropiadas de antibióticos orales en 2015, lo que representa el 24 % de las ventas totales de antibióticos de 74 400 millones de unidades (tanto por vía oral como parenteral) en la base de datos. Los cinco antibióticos principales del Grupo de acceso apropiados para niños según la cantidad que se vendió fueron la amoxicilina, la amoxicilina con ácido clavulánico, la trimetoprima-sulfametoxazol, la cefalexina y la ampicilina. El porcentaje de los cinco antibióticos más vendidos para su uso como jarabe varió entre el 42 % y el 99 %. Los comprimidos dispersables representaron solo el 22 % de todas las formulaciones pediátricas orales apropiadas que se vendieron y representaron solo el 15 % de las ventas de los 10 antibióticos del Grupo de acceso elegidos de la lista modelo pediátrica.

**Conclusión:**

La mayoría de los antibióticos orales apropiados para los niños no se vendieron en forma de comprimidos dispersables en 2015 a nivel mundial, como recomendó la OMS. Es evidente la necesidad de desarrollar nuevas formas sólidas de antibióticos adecuados para su uso pediátrico.

## ملخص

الغرض استقصاء أنماط الاستهلاك الدولي للتركيبات المناسبة للمضادات الحيوية للأطفال عن طريق الفم، حسب نوع التركيب، مع التركيز على الأقراص القابلة للانتشار، وباستخدام البيانات من قاعدة بيانات المبيعات العالمية.

الطريقة تم الحصول على بيانات مبيعات المضادات الحيوية لعام 2015، والتي تغطي 74 دولة ومجموعات دول إقليمية، من قاعدة بيانات المبيعات الصيدلانية ®MIDAS، والتي تشمل عينات من تجار الجملة وتجار التجزئة للصيدليات. كان التركيز على مبيعات التركيبات المناسبة عن طريق الفم للأطفال من المضادات الحيوية المتاحة في المتناول في القائمة النموذجية للأدوية الأساسية للأطفال التابعة لمنظمة الصحة العالمية (WHO) عام2017. يتم التعبير عن أحجام المبيعات باستخدام وحدة قياسية (أي قرص واحد، أو كبسولة واحدة، أو أمبول واحد، أو قنينة واحدة، أو 5 مل من السائل). وتم تحليل المبيعات تبعاً للمضاد الحيوي، ومنطقة منظمة الصحة العالمية، وتركيب المضاد الحيوي.

النتائج على الصعيد العالمي، تم بيع 17.7 مليار وحدة قياسية من تركيبات المضادات الحيوية المناسبة للأطفال عن طريق الفم عام 2015، وهو ما يمثل 24٪ من إجمالي مبيعات المضادات الحيوية التي تبلغ 74.4 مليار وحدة (سواء عن طريق الفم أو بالحقن) في قاعدة البيانات. كانت المضادات الحيوية الخمسة الأولى، المتاحة في المتناول والمناسبة للأطفال، حسب حجم المبيعات هي: أموكسيسيلين، وأموكسيسيلين مع حمض كالفولانيك، وترايميثوبريم سلفاميثوكسازول، وسيفاليكسين، وأمبيسيلين. وتراوحت نسبة الخمسة الأكثر مبيعاً للاستخدام كشراب بين 42٪ و99٪. بينما كانت الأقراص القابلة للانتشار تمثل 22٪ فقط من جميع مبيعات التركيبات المناسبة للأطفال عن طريق الفم، وشكلت 15٪ فقط من مبيعات 10 من المضادات الحيوية المتاحة في المتناول، المختارة في القائمة النموذجية للأطفال.

الاستنتاج لم يتم بيع معظم المضادات الحيوية المناسبة للأطفال عن طريق الفم على مستوى العالم كأقراص قابلة للانتشار في عام 2015، كما أوصت منظمة الصحة العالمية (WHO). هناك حاجة واضحة لأشكال صلبة جديدة من المضادات الحيوية المناسبة للاستخدام بواسطة الأطفال.

## 摘要

**目标:**

旨在利用全球销售数据库的数据，按剂型调查儿童口服抗生素的国际消费模式，重点关注分散片。

**方法:**

选取 MIDAS® 医药销售数据库 2015 年涵盖的 74 个国家和地区国家组的抗生素销售数据，其中包括医药批发商和零售商的样本。重点关注 2017 年世界卫生组织（世卫组织）*《儿童基本药物标准清单》*中儿童口服抗生素制剂的销售情况。使用标准单位（即一片、胶囊、安瓶或小瓶或 5 毫升液体）表示销售量。根据抗生素、世界卫生组织地区和抗生素剂型对销售情况进行分析。

**结果:**

2015 年，全球共销售 177 亿个标准单位的儿童口服抗生素制剂，占数据库 744 亿个单位（口服和非口服）抗生素总销量的 24%。销售量最高的五种儿童口服抗生素制剂是阿莫西林、阿莫西林-克拉维酸、甲氧苄啶-磺胺甲恶唑、头孢氨苄和氨苄西林。在销售量最高的五种制剂中，用于制作糖浆的比例在 42%-99% 之间。分散片仅占所有儿童口服制剂销售量的 22%，在儿童清单选定的 10 种儿童抗生素销售量中仅占 15%。

**结论:**

2015 年全球大部分的儿童口服抗生素未按照世界卫生组织的建议作为分散片出售。显然，当前仍十分有必要研制出适合儿童使用的新型固体抗生素。

## Резюме

**Цель:**

Исследование глобальных моделей потребления пероральных детских лекарственных форм антибиотиков с уделением особого внимания диспергируемым таблеткам с помощью сведений из глобальной базы данных о продажах.

**Методы:**

Данные о продажах антибиотиков в 74 странах мира и региональных группах стран за 2015 год были получены из базы данных продаж фармацевтической продукции MIDAS^®^, содержащей данные по оптовым и розничным аптекам. Основное внимание уделялось продажам пероральных детских лекарственных форм антибиотиков из группы массовой доступности (согласно *Примерному перечню *Всемирной организации здравоохранения (ВОЗ)* основных лекарственных средств для детей* на 2017 год). Объемы продаж были выражены в стандартных единицах (то есть одна таблетка, капсула, ампула или флакон либо 5 мл жидкости). Анализ продаж осуществлялся по антибиотикам, региону ВОЗ и лекарственным формам.

**Результаты:**

В 2015 году в мире было продано 17,7 млрд стандартных единиц пероральных лекарственных форм антибиотиков, приемлемых для детей, что составляет 24% от общего объема продаж антибиотиков (74,4 млрд единиц как пероральных, так и парентеральных лекарственных форм), зарегистрированного в базе данных. В пятерку лидеров по объемам продаж детских антибиотиков из группы массовой доступности вошли амоксициллин, амоксициллин + клавулановая кислота, триметоприм/сульфаметоксазол, цефалексин и ампициллин. Доля продаж пяти лидеров в виде сиропа варьировалась от 42 до 99%. Диспергируемые таблетки составляли лишь 22% от общего объема продаж пероральных детских лекарственных форм и всего 15% от общего объема продаж 10 выбранных антибиотиков из группы массовой доступности Примерного перечня основных лекарственных средств для детей.

**Вывод:**

В 2015 году во всем мире большинство приемлемых для детей пероральных лекарственных форм антибиотиков не продавались в форме диспергируемых таблеток, как это было рекомендовано ВОЗ. Существует явная необходимость в выпуске новых твердых лекарственных форм антибиотиков, подходящих для применения у детей.

## Introduction

The principle aim of the sustainable development goal 3 is to improve equity of access to health care, which includes ending preventable deaths in neonates and children younger than 5 years (or achieving a reduction to at most 25 deaths per 1000 live births) by 2030.^1^ More than half of deaths in children younger than 5 years are estimated to be due to infectious disease, with pneumonia being the leading cause.^2^ In addition, the authors of the World Health Organization’s *WHO* *Model list of essential medicines for children*,^3^ which was first published in 2007, state that reliable access to key medicines is vital for ensuring basic health-care needs are met.

Recently WHO announced its first specific target for combating antimicrobial resistance:^4^ Sixty percent of all antibiotics consumed in individual countries should come from the Access group of antibiotics (i.e. first- or second-choice empirical treatments for selected syndromes).^3^^,^^5^ The classification of antibiotics into Access, Watch and Reserve categories (the AWaRe classification) was introduced by WHO in 2017 to combat antimicrobial resistance. In children, the main Access antibiotic is amoxicillin because WHO guidelines currently recommend oral amoxicillin as first-line therapy for suspected lower respiratory tract infection in those younger than 5 years.^6^ Although differentiating viral and bacterial respiratory tract infections in children remains complex, a substantial proportion of cases of bacterial childhood pneumonia worldwide are still not treated promptly with antibiotics. In addition, access to drugs on the model list for children remains limited in low- and middle-income countries.^7^

To explore current patterns of antibiotic use in children, we investigated sales of child-appropriate antibiotic formulations. We defined a child-appropriate formulation as an oral formulation specifically designed for easy use in children that was either: (i) an oral liquid formulation; or (ii) an oral solid formulation that is dispersed in water or becomes liquid upon swallowing. Adult tablets were not included, as it has been shown that crushing (i.e. compounding) tablets to give to children can result in underdosing.^8^ Consumption of oral formulations was chosen as a proxy for child antibiotic use for several reasons. First, caregivers’ compliance with administering prolonged courses of oral antibiotics has been shown to correlate with the availability of liquid formulations.^9^ Second, data from studies of the palatability of different formulations of paediatric antiretroviral therapy indicate that most drugs consumed by children younger than 5 years are in a non-solid form.^10^^,^^11^ Third, the number of prescriptions for antibiotics dispensed in a liquid formulation has been shown to correlate with the number of children in a population.^12^

Amoxicillin, for example, is available in several child-appropriate formulations, including granules, dispersible tablets and oral suspensions or syrups. Amoxicillin dispersible tablets have many advantages over oral suspensions and syrups: (i) they cost less; (ii) their volume is smaller because the weight per unit dose is lower, which means that less storage space is required in the supply chain; (iii) dosing is simpler; and (iv) refrigeration is unnecessary.^13^ Moreover, amoxicillin in dispersible tablet form was identified as one of 13 overlooked life-saving commodities by the United Nations Commission on Life-Saving Commodities in Women and Children.^14^ The commission promotes access to these commodities, which can prevent premature death in women and children, by encouraging innovations in product design and increased production. Over the past 5 years, the United Nations Children's Fund (UNICEF) has supported the large-scale procurement of amoxicillin dispersible tablets and the supply has increased more than 10-fold.^13^

In 2018, WHO published the first method for a global antibiotic consumption surveillance programme,^15^ which acknowledged that estimating paediatric antibiotic consumption was difficult because standardized daily defined dosages are not available for children. Currently there are no published estimates of the total annual volume of antibiotics sold for paediatric use that can be used to derive the proportion sold as dispersible tablets and that can provide some estimate of the potential unmet need. Further, no published data exist on the consumption of dispersible tablets of antibiotics on the *WHO Model list of essential medicines for children* or on how consumption patterns of paediatric antibiotic formulations vary between WHO regions.^16^ The demand and supply of child-appropriate formulations are affected by many factors; for example, the supply of trimethoprim–sulfamethoxazole is subsidized by the Global Fund to Fight AIDS, Tuberculosis and Malaria in some regions.^17^

Data from the MIDAS^®^ database (IQVIA, Durham, United States of America) on global pharmaceutical sales have previously been used to provide a broad overview of international trends in antibiotic sales and, by proxy, antibiotic consumption.^18^^–^^20^ The aim of our study was to investigate global consumption patterns of child-appropriate oral formulations of antibiotics by formulation type, with a focus on dispersible tablets. We used formulation-specific sales data from the MIDAS^®^ database as a proxy for paediatric antibiotic consumption and combined consumption data with demographic data from the United Nations to derive consumption per child.^19^^,^^21^^,^^22^

## Methods

We obtained antibiotic sales data for 2015 from the MIDAS^®^ pharmaceutical sales database, which includes information on sales from samples of pharmacy wholesalers and retailers in participating countries. The data included sales volumes (in standard units) through specific channels to pharmacies, drug outlets and hospitals, with the relative contributions of different channels to total sales varying between countries. No information on dosing or clinical indications was available to us. Data collected from Europe and North America were biased heavily towards procurement for public health-care systems, whereas data for other regions largely originated from the private sector. Although analyses show that market coverage can vary from 20 to 100% in contributing countries,^18^^–^^21^ the MIDAS^®^ database provides one of the most comprehensive insights into global pharmaceutical sales patterns and has made a substantial contribution to the pharmacoepidemiology literature. Furthermore, data validation and quality assurance are carried out annually by IQVIA.^23^

Pharmaceutical sales data are reported in standard units, where a standard unit is equivalent to a single dose by volume (not by quantity of active ingredient): it can be one tablet, capsule, ampoule or vial or 5 mL of liquid.^19^^,^^21^ For example, a standard course of amoxicillin administered as one 5-mL teaspoon or one dispersible tablet given twice daily for 5 days would total 10 standard units. However, we were unable to determine from the sales data whether child-appropriate syrup formulations were actually consumed by children as they may also have been used by elderly people or by other adults unable to swallow solid formulations. Our primary data analysis included sales data on oral and parenteral antibiotics sold in 93 different formulations across 74 countries and regional country groups. A secondary analysis focused on antibiotics on the 2017 *WHO Model list of essential medicines for children*. The database did not include information on nitrofurantoin.

We identified 22 child-appropriate oral antibiotic formulations covered by the MIDAS^®^ database for inclusion in our study by independently selecting new form codes (available from the data repository).^24^ Formulation data held in the database are classified using new form codes, which were introduced by the European Pharmaceutical Market Research Association in 1986, in parallel with the Anatomical Therapeutic Chemical classification system.^25^ Countries included in the database were grouped by WHO region: (i) two countries and one regional group in the African Region; (ii) 13 countries and one regional group in the Region of the Americas; (iii) six countries in the South-East Asia Region; (iv) 33 countries in the European Region; (v) nine countries in the Eastern Mediterranean Region; and (vi) 10 countries in the Western Pacific Region. In the African Region, 10 countries were grouped as Francophone West Africa (i.e. Benin, Burkina Faso, Cameroon, Côte d'Ivoire, Democratic Republic of the Congo, Gabon, Guinea, Mali, Senegal and Togo) and, in the Region of the Americas, six countries were grouped as Central America (i.e. Costa Rica, El Salvador, Honduras, Guatemala, Nicaragua and Panama). The top five child-appropriate antibiotic formulations by sales volume in each WHO region were identified and the proportion of these top five formulations sold as dispersible tablets was calculated for each region.

Of the 22 child-appropriate oral antibiotic formulations identified, 10 were Access antibiotics that were listed as a first- or second-line choice for treating clinical indications in the model list for children (Box 1).^16^ We analysed their sales in detail. Several of these formulations require preparation before use, such as the addition of water; their new form code either starts with the letter D or E or is AAH or AAK.^25^ We also used these codes to determine the proportion of each antibiotic that was sold in the form of a dispersible tablet, solid tablet, liquid or granule or in another form.

Box 1 **Selected oral Access antibiotics, study of global consumption of child-appropriate antibiotic formulations, 2015**

**Beta-lactam antibiotics:**

Amoxicillin; Ampicillin; Cefalexin; Cloxacillin; and Penicillin V

**Other antibiotics:**

Chloramphenicol; Clindamycin; Doxycycline; Metronidazole; and Trimethoprim–sulfamethoxazole

Note: All Access antibiotics (i.e. first- or second-choice empirical treatments for selected syndromes) listed were all included in the World Health Organization’s *WHO* *Model list of essential medicines for children*.^3^

We obtained estimates of the total population of each country from the MIDAS^®^ data set and estimates of the population of children younger than 5 years from the United Nations Population Division data for 2015.^22^ Using these figures, we were able to calculate the number of standard units of each child-appropriate formulation sold per child younger than 5 years in each country. These were regarded as minimum consumption estimates because other important sources of antibiotics, for example donors, were not included in the MIDAS^®^ data set. All statistical analyses were carried out using RStudio v. 1.0.143 (RStudio, Boston, USA).

## Results

In 2015, 17.7 billion standard units of the 22 selected child-appropriate oral antibiotic formulations were sold in countries contributing to the MIDAS^®^ data set out of a total of 74.4 billion standard units of all antibiotic formulations (i.e. oral and parenteral). Hence, child-appropriate oral formulations accounted for 24% (17.8 billion/74.4 billion) of global antibiotic sales. Dispersible tablet sales accounted for 22% (4.0 billion/17.8 billion) of all child-appropriate oral formulation sales, whereas 77% (13.6 billion/17.8 billion) were in granule, powder or another form. The remainder were tablets that could be chewed or disperse in the mouth.

**Fig. 1:**
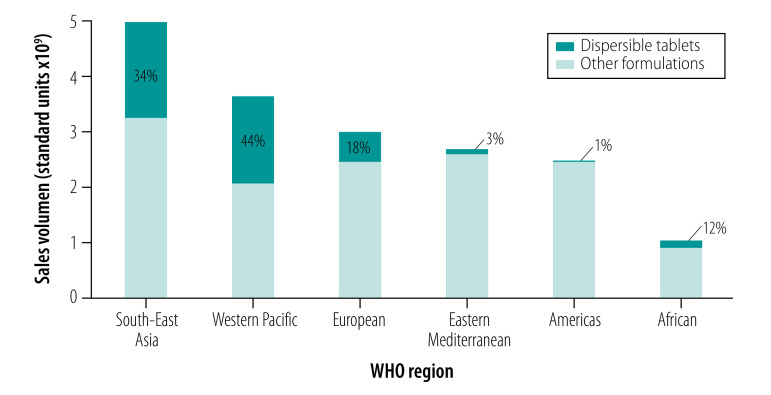
Sales of child-appropriate oral antibiotics and the proportion sold as dispersible tablets, by WHO region, 2015

Fig. 1 shows the total volume of sales of the 22 selected child-appropriate oral antibiotic formulations in the six WHO regions and the percentage of those sales accounted for by dispersible tablets. The total sales volume was highest in the South-East Asia Region and the proportion of sales attributable to dispersible tablets was highest in the South-East Asia and Western Pacific Regions (34%; 1.7 billion/5.0 billion; and 44%; 1.6 billion/3.6 billion, respectively). The uptake of dispersible tablets was notably lower in the Region of the Americas and the Eastern Mediterranean Region: the proportion of sales attributable to dispersible tablets was 1% (13.0 million/2.5 billion) and 3% (7.3 million /2.7 billion) in these regions, respectively. Details of the five most commonly used child-appropriate antibiotic formulations in each WHO region are available from the data repository.^24^ In particular, cefixime was widely used in the Western Pacific and South-East Asian Regions.

WHO: World Health Organization.

Notes: Sales of 22 child-appropriate antibiotic formulations recorded on the MIDAS^®^ database were included. A standard unit is equivalent to a single dose by volume: it can be one tablet, capsule, ampoule or vial or 5 mL of liquid.

**Fig. 2:**
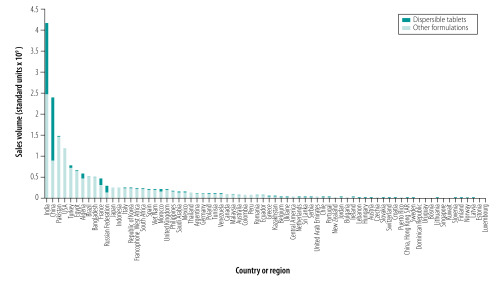
Sales of child-appropriate oral antibiotics and the proportion sold as dispersible tablets, by country or regional country group, 2015

**Fig. 3:**
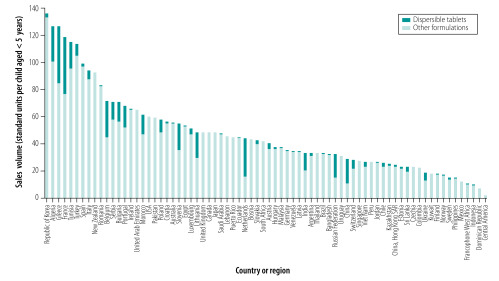
Sales of child-appropriate oral antibiotics per child younger than 5 years and the proportion sold as dispersible tablets, by country or regional country group, 2015

Fig. 2 shows the sales of the 22 selected child-appropriate oral antibiotic formulations in individual countries in 2015 and the percentage of those sales attributable to dispersible tablets. The highest sales of dispersible tablets were in China and India. Fig. 3 shows the sales per child in individual countries and the proportion of sales per child attributable to dispersible tablets. The median sales per child was 43 standard units (interquartile range: 26–59). The proportion of sales per child attributable to dispersible tablets varied widely between countries: the highest proportions were found in Algeria, Belgium, France, Greece and the Netherlands.

SAR: Special Administrative Region.

Notes: Sales of 22 child-appropriate antibiotic formulations recorded on the MIDAS^®^ database were included. A standard unit is equivalent to a single dose by volume: it can be one tablet, capsule, ampoule or vial or 5 mL of liquid. Francophone West Africa included Benin, Burkina Faso, Cameroon, Côte d'Ivoire, Democratic Republic of the Congo, Gabon, Guinea, Mali, Senegal and Togo and Central America included Costa Rica, El Salvador, Honduras, Guatemala, Nicaragua and Panama.

SAR: Special Administrative Region.

Notes: Sales of 22 child-appropriate antibiotic formulations recorded on the MIDAS^®^ database were included. A standard unit is equivalent to a single dose by volume: it can be one tablet, capsule, ampoule or vial or 5 mL of liquid. Francophone West Africa included Benin, Burkina Faso, Cameroon, Côte d'Ivoire, Democratic Republic of the Congo, Gabon, Guinea, Mali, Senegal and Togo and Central America included Costa Rica, El Salvador, Honduras, Guatemala, Nicaragua and Panama.

Table 1 shows that approximately 10.1 billion standard units of child-appropriate oral formulations of the 10 Access antibiotics on the 2017 *WHO Model list of essential medicines for children* were sold globally in 2015, which accounted for 56% (10.1 billion/17.8 billion) of all child-appropriate oral antibiotics formations sold. The majority were taken in liquid form, mostly consumed as a syrup: 67% (6.8 billion/10.1 billion) were sold as a powder and required the addition of water to make them ready for consumption, whereas 15% (1.5 billion/10.1 billion) were sold as a ready-made syrup. Tablets that had to be dispersed in water accounted for only 16% (1.6 billion/10.1 billion) of sales. Overall, 78% (7.8 billion/10.1 billion) of sales of child-appropriate formulations of the 10 Access antibiotics were accounted for by amoxicillin and amoxicillin with clavulanic acid, generally for consumption as a liquid. Trimethoprim–sulfamethoxazole, chloramphenicol and metronidazole were predominantly sold as ready-made syrups. In contrast, the non-β-lactams doxycycline and clindamycin were commonly sold as dispersible tablets.

**Table 1:** Global sales of oral Access antibiotics^a^ in child-appropriate formulations, by formulation, 2015

Antibiotic	Sales volume,^b^ no. of standard units^c^ in millions (%)					
	Total	Tablet	Dispersible tablet	Granule or powder	Ready-made syrup	Syrup (sold in dry form)
Amoxicillin	4 256.0 (100)	55.6 (1)	918.1 (22)	71.8 (2)	299.9 (7)	2910.6 (68)
Amoxicillin with clavulanic acid	3 550.2 (100)	31.0 (1)	318.8 (9)	0.0 (0)	85.7 (2)	3114.8 (87)
Trimethoprim–sulfamethoxazole	875.3 (100)	0.0 (0)	0.6 (< 1)	5.7 (< 1)	860.8 (98)	8.2 (1)
Cefalexin	650.7 (100)	< 0.1 (< 0.1)	64.9 (10)	2.5 (< 1)	120.0 (18)	463.1 (71)
Ampicillin	251.9 (100)	0.0 (0)	145.6 (58)	0.0 (0)	15.4 (6)	90.2 (36)
Clindamycin	190.5 (100)	0.0 (0)	114.9 (60)	< 0.1 (< 1)	0.8 (< 1)	74.8 (39)
Penicillin V	189.1 (100)	0.0 (0)	0.3 (< 1)	0.0 (0)	48.2 (26)	140.5 (74)
Chloramphenicol	100.3 (100)	0.0 (0)	0.7 (< 1)	0.0 (0)	99.6 (99)	< 0.1 (< 1)
Doxycycline	58.0 (100)	< 0.1 (< 1)	54.8 (95)	0.0 (0)	2.3 (4)	0.7 (1)
Cloxacillin	21.6 (100)	0.0 (0)	0.0 (0)	0.0 (0)	2.3 (11)	19.3 (89)
Metronidazole	1.2 (100)	0.0 (0)	0.0 (0)	0.0 (0)	1.2 (100)	< 0.1 (0)
**Total**	**10 145.0 (100)**	**86.6 (< 1)**	**1619.0 (16)**	**80.0 (< 1)**	**1536.3 (15)**	**6823.0 (67)**

^a^ All Access antibiotics listed are first- or second-choice empirical treatments for selected syndromes and are included in the World Health Organization’s *WHO* *Model list of essential medicines for children*.^3^

^b^ Reported in the MIDAS^®^ database.

^c^ A standard unit is equivalent to a single dose by volume: it can be one tablet, capsule, ampoule or vial or 5 mL of liquid.

## Discussion

Our analysis shows that less than a quarter of child-appropriate oral antibiotic formulation sales in 2015 globally were in the form of dispersible tablets. Moreover, two thirds were still sold as liquid formulations, which indicates that, by 2015, liquids and powders requiring reconstitution had not fully replaced dispersible tablets, as recommended by WHO.^6^ Over three quarters of sales of the 10 child-appropriate formulations of Access antibiotics we investigated were of amoxicillin or amoxicillin with clavulanic acid.

The wide variation we found in the per-child consumption of child-appropriate antibiotic formulations (Fig. 3) may be the result of several factors. In particular, further research is needed to explore whether this variation reflects the appropriate clinical use of antibiotics (appropriateness could not be determined from MIDAS^®^ data), the need for greater access to child-appropriate formulations or other system factors.

International comparisons of paediatric antibiotic consumption face considerable challenges. Defining the appropriate sampling frame or the system level at which any measurement of consumption can be considered representative of a country can be difficult. Many previous analyses of primary care or outpatients have assessed prescriptions rather than consumption,^26^^,^^27^ which reflects the difficulty of measuring consumption. Nevertheless, a recent analysis of WHO data collected using service provision assessments and service availability and readiness assessments confirms that the paediatric use of antibiotics in primary care is high for all clinical infections.^28^

For comparison, the MAL-ED study examined the number of courses of antibiotics used in the community in 2134 children younger than 2 years in eight countries: one site each in Bangladesh, Brazil, India, Nepal, Pakistan, Peru, South Africa and the United Republic of Tanzania.^29^ Bangladesh and Pakistan had the highest overall antibiotic consumption per child (i.e. over 10 courses per year), whereas Brazil and South Africa had the lowest, with a 10-fold difference. In our study, we also found that of these eight countries, Pakistan had the highest annual consumption per child of child-appropriate antibiotic formulations (Fig. 2). However, Bangladesh, Brazil and South Africa had very similar levels of consumption. The difference between the two studies was possibly due to differences in data collection and study population: (i) the MAL-ED study conducted active surveillance of antibiotic use, whereas the MIDAS^®^ database recorded sales; and (ii) the MAL-ED study used defined study sites and age groups, whereas the MIDAS^®^ database had a varying level of national coverage and used formulation-specific sales data as a proxy for consumption in children.

Our study aimed to provide a broad overview of the absolute volume of oral antibiotics sold in child-appropriate formulations across countries. However, the volumes reported here should be regarded as minimum estimates because the MIDAS^®^ database does not capture data from some important global organizations. For example, UNICEF procured 193 million amoxicillin dispersible tablets in 2015,^13^ a substantial increase from 14 million in 2011. A complete analysis of the global market for oral antibiotics for children would need to include direct procurement by the public sector, donations from global organizations and sales in the informal sector. Our analysis should be considered as providing a baseline for paediatric antibiotic sales before implementation of the revised 2017 *WHO Model list of essential medicines for children*, in which the AWaRe classification was introduced.^3^ Previously we investigated time trends in the use of child-appropriate antibiotic formulations;^21^ although we found a slight change over time, no major fluctuations in global use from year to year were identified.

There is no clear evidence in the literature indicating, which antibiotic formulation is easiest to administer to children, most acceptable to children and families, and results in the best clinical outcomes.^30^ The key advantages of dispersible tablets are programmatic: (i) they require less space on transportation; (ii) they can be stored for prolonged periods; and (iii) their dosing is simpler than for liquid formulations or powders, which require doses to be measured before reconstitution. However, dispersible tablets have some disadvantages, such as palatability. In addition, they require a clean water source (a typical tablet needs 5 to 10 mL of water for dispersion) and caregivers must spend time waiting for the tablet to dissolve. In addition, an estimated 25% of the global disease burden in children younger than 5 years is attributable to unsafe water, poor sanitation or inadequate hygiene,^31^ which are being assessed by UN-Water and WHO as part of the sustainable development goal programme.^32^ Nevertheless, recent evidence suggests that minitablets are tolerated by children as young as 2 months of age.^30^ Future research could focus on the development of oral-dispersible tablets or other forms of antibiotics that do not require water for administration, such as drugs that can be delivered through the transmucosal route.^33^ Such developments could be facilitated by recent WHO initiatives, such as the Global Accelerator for Paediatric Formulations,^34^ which is working in collaboration with the WHO Department of Essential Medicines and Health Products and other key stakeholders, including the Global Antibiotic Research and Development Partnership.

We acknowledge that the main limitation of our study is its reliance on sales data from varying sources. However, the MIDAS^®^ database provides detailed information on wholesale pharmacy sales of paediatric antibiotics in many countries and we know of no other database that can be used to compare the sales of child-appropriate antibiotic formulations internationally. Another limitation is that the MIDAS^®^ database has a varying level of coverage of the public sector and of different supply channels, depending on region and the local health-care delivery structure. As paediatric medicines may be prioritized by publicly-funded health-care systems, it is difficult to predict, which supply channels will provide the most accurate data on child-appropriate formulation sales. In addition, data on sales, particularly of dispersible tablets, in individual WHO regions may be dominated by a few large countries, such as China, India or the United States (Fig. 2).

In conclusion, our study used the largest available pharmaceutical sales data set to provide a baseline for the consumption of child-appropriate oral antibiotic formulations globally in 2015. We found that most of child-appropriate formulations sold globally in 2015 were not in a dispersible tablet form. Future efforts should focus on developing improved solid forms of antibiotics suitable for use in children and encouraging their widespread use.^35^
